# Amino acid nitrogen and carbon isotope data: Potential and implications for ecological studies

**DOI:** 10.1002/ece3.8929

**Published:** 2022-06-02

**Authors:** Hee Young Yun, Thomas Larsen, Bohyung Choi, Eun‐Ji Won, Kyung‐Hoon Shin

**Affiliations:** ^1^ Deparment of Marine Science and Convergent Technology Hanyang University Ansan Korea; ^2^ 442078 Department of Archaeology Max Planck Institute for the Science of Human History Jena Germany; ^3^ 183695 Inland Fisheries Research Institute National Institute of Fisheries Science Geumsan‐gun Korea

**Keywords:** amino acid‐specific isotope analysis, biomarkers, diet estimate, isotope differentiation, trophic enrichment, trophic interaction

## Abstract

Explaining food web dynamics, stability, and functioning depend substantially on understanding of feeding relations within a community. Bulk stable isotope ratios (SIRs) in natural abundance are well‐established tools to express direct and indirect feeding relations as continuous variables across time and space. Along with bulk SIRs, the SIRs of individual amino acids (AAs) are now emerging as a promising and complementary method to characterize the flow and transformation of resources across a diversity of organisms, from microbial domains to macroscopic consumers. This significant AA‐SIR capacity is based on empirical evidence that a consumer's SIR, specific to an individual AA, reflects its diet SIR coupled with a certain degree of isotopic differences between the consumer and its diet. However, many empirical ecologists are still unfamiliar with the scope of applicability and the interpretative power of AA‐SIR. To fill these knowledge gaps, we here describe a comprehensive approach to both carbon and nitrogen AA‐SIR assessment focusing on two key topics: pattern in AA‐isotope composition across spatial and temporal scales, and a certain variability of AA‐specific isotope differences between the diet and the consumer. On this basis we review the versatile applicability of AA‐SIR to improve our understanding of physiological processes as well as food web functioning, allowing us to reconstruct dominant basal dietary sources and trace their trophic transfers at the specimen and community levels. Given the insightful and opportunities of AA‐SIR, we suggest future applications for the dual use of carbon and nitrogen AA‐SIR to study more realistic food web structures and robust consumer niches, which are often very difficult to explain in nature.

## Graphical abstract

Stable isotope analysis of individual amino acids (AAs) in natural abundance is now emerging as a promising and complementary method to characterize the flow and transformation of resources across a diversity of organisms, from microbial domains to macroscopic consumers. Many empirical ecologists are still unfamiliar with the scope of applicability and the interpretative power of AA‐stable isotope ratios. Here, we describe how both carbon and nitrogen AA‐SIR assessment can help our understanding of direct and indirect feeding relations as continuous variables across time and space in ecological and physiological background.

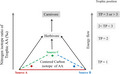

## 1 INTRODUCTION

Ecosystems and life on Earth are subject to global environmental stressors resulting from human activities, including overfishing, invasive species, species loss, and habitat degradation. These natural and anthropogenic environmental stressors affect the number and portions of basal components, such as living autotrophs (to support “green” food webs) and decomposed organic matter (OM) (to support “brown” food webs or “microbial loops”; Quévreux et al., 2021; Wolkovich et al., 2014; Zou et al., 2016). These ongoing global environmental changes have also led to ecosystem‐wide impacts, including trophic connections, numbers of trophic levels (Lynam et al., 2017; Maureaud et al., 2017), the consumer population abundance, and omnivory levels (Gibert, 2019). However, it is not simple to say how a dynamic climate change is related to ecosystem‐wide impacts in the face of complex temporal and environmental variations, inherently combined with biogeochemical and ecological processing (Hussey et al., 2014; McMeans et al., 2015). A more elaborate methodology could enable us to assess environmental risks and vulnerabilities (e.g., natural and/or anthropogenic impacts) and to develop conservation management plans.

Naturally occurring stable isotope variations, particularly those of carbon (C) and nitrogen (N), are powerful tools for describing trophic interactions, food web structures, and biogeochemical processes. This is due to (1) the fixed isotope signals in the sources, which are isotopically distinct, and (2) the predictable isotope fractionation factor at the organism and environment levels. The isotope fractionation factor is the factor by which the abundance ratio of two isotopes will change during a physiochemical process. The isotope fractionation factors occur due to reaction rate differences between lighter (e.g., ^12^C and ^14^N) and heavier isotopes (e.g., ^13^C and ^15^N) during physicochemical and biogeochemical process, which leaves the consumer with a higher ratio of heavier than light isotopes in their diet (or source element) (i.e., Fry, 2006; Fry & Carter, 2019; Martínez del Rio et al., 2009). It has long been established that consumers become more enriched with heavy isotopes than with light isotopes relative to their dietary resources; for C, the isotope difference is small at 0–1‰, and for N it is significantly higher at 2–4‰ (DeNiro & Epstein, 1978, 1981; Fry, 2006; Minagawa & Wada, 1984). Thus, stable isotope ratios (SIR) can provide the evidence related to the trophic connections as well as trophic hierarchy between consumer and potential (or actual) dietary resource. The applications of SIR are extended to estimate nutritional or basal resources (also referred to as food web end‐members or baselines) as well as the varying reliance on those different resources quantitatively for consumers which support local food webs (Middelburg, 2014; Newsome et al., 2007).

Many studies assert that consumers’ isotope values (i.e., the whole‐tissue parts) are confounded by variable temporal and spatial traits (reviewed in Shipley & Matich, 2020), inducing isotope variations at the base of local food webs. For instance, stable C isotope ratios (^13^C: ^12^C, represented by δ^13^C) are useful to separate photosynthetic pathways (C_3_, C_4_, and CAM plants), inorganic sources (e.g., CO_2_ and bicarbonate), but also can be affected by environmental change (Zhang et al., 2019) as well as regional variances (e.g., marine vs. terrestrial, benthic vs. pelagic algae, nearshore vs. offshore productivity, and high vs. low latitude; Glibert et al., 2019, references therein). Similarly, stable N isotope ratios (^15^N: ^14^N, represented by δ^15^N) can differ among sources such as N_2_, NH_4_^+^, NO_3_^−^, and amino acids (AAs) as well as other environmental characteristics of locations or sites (e.g., precipitation, altitude, temperature, and salinity; Hyodo, 2015; Kelly, 2000). It indicates that C and N bulk isotope ratios would be variable in consumers and producers, as do baseline isotope ratios vary over time and regions (Drobnitch et al., 2018; Zhang et al., 2021). Thus, it is notably important for assessing correct trophic interactions in local food webs to distinguish effect of dietary sources and/or environmental differences on consumer isotope ratios (Blattmann & Ishikawa, 2020; Middelburg, 2014).

**FIGURE 1:**
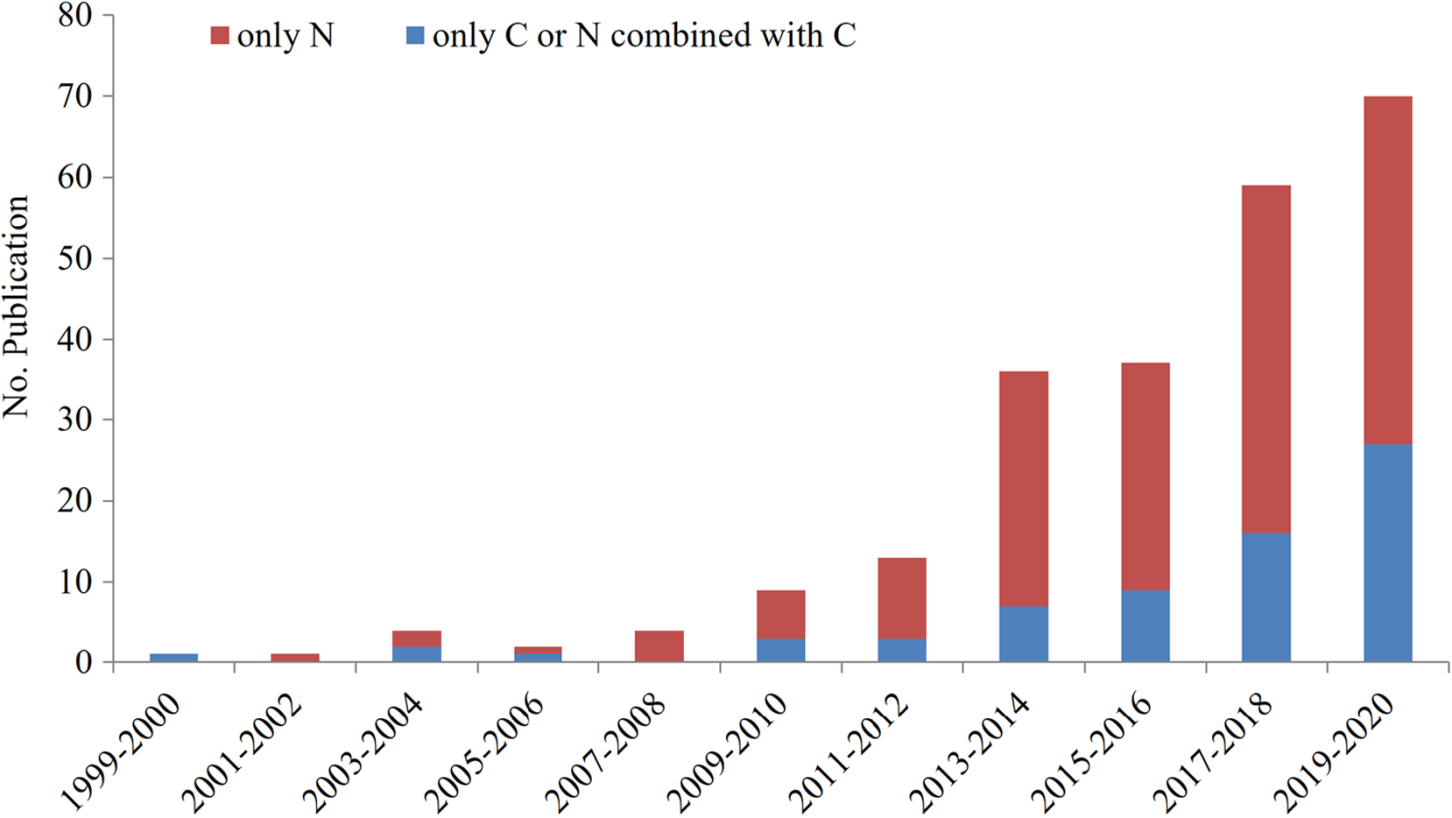
Trend of numbers of trophic ecology‐based publications that have employed compound‐specific stable isotope analysis till 2020, which include all peer‐reviewed publications in the Web of Science database published before this date. We conducted this review by first searching for amino acid isotope and narrowed the search terms to food web, resource, diet, and consumer. We then removed all publications that were listed twice, which resulted in a total of 267 references

Ecologists have considered amino acid (AA)‐SIR as a complementary and straightforward approach of bulk SIR analysis (Table 1). AAs, the building blocks of protein as essential macronutrients, are the persistent and abundant organic compounds (Ruess & Müller‐Navarra, 2019), and are found in diverse sample types from biological samples as well as environmental samples. Thanks to advances in continuous‐flow isotope analyses of single compounds in the late 1990s, applications of AA δ^15^N and AA δ^13^C have become more widespread and common over the last decades (Figure 1). AA‐SIR analysis has been extensively applied to elucidate trophic connections from field as well as lab‐simulated experiments, designed to examine simplified food chains (e.g., consumer–direct diet interactions) and multi‐trophic level (e.g., producer–herbivore–carnivore). There have been recent reviews of AA‐SIR applications in ecological and geochemical studies (Ishikawa, 2018; McMahon & McCarthy, 2016; Ohkouchi et al., 2015, 2017; Whiteman et al., 2019) and the biochemical background in AA‐SIR isotope variability (Ohkouchi et al., 2015; Whiteman et al., 2019) and analytic methods (Ohkouchi et al., 2017). These influential reviews were mostly emphasized to trace a source and its trophic transfer with AA δ^15^N analysis, while relatively less focused on the role of AA δ^13^C approaches. In contrast, this review will coordinate and synthesize the application scope of AA δ^15^N as well as AA δ^13^C approach, which reconstruct various types of trophic links from single source to multiple sources (e.g., mixed plant/detrital OM and terrestrial/aquatic resources) in ecosystems. Additionally, this review will highlight some of the challenges and potential future directions to collaborate AA δ^15^N and AA δ^13^C composition for better understanding of complex food webs and consumer niches in local environments. Firstly, we outline the AA‐SIR variability patterns and their physiological/ecological basis, before turning to the topic of coupling C and N trophic channels.

**TABLE 1:** Comparing strategies of traditional bulk and amino acids isotope approaches for diet tracing and food web reconstruction

Major themes	Bulk δ^13^C and δ^15^N analysis	Amino acid δ^15^N analysis	Amino acid δ^13^C analysis
Diet resolution	If used to two isotopes, max. 3 items	Assumed limited number (e.g., terrestrial plant or aquatic algae)	3–6 phylogenetically separated groups in autotrophs
Isotopic variability by non‐dietary factor	Variable due to difference in basal source across sites	Variable, similar to bulk N isotope	Robust for essential AAs after normalization
Trophic discrimination factor (TDF)	Variable (e.g., 3.4 ‰ for δ^15^N, 0.4 ‰ for δ^13^C, Post 2002)	Variable depends on AAs (e.g., 8.0 ‰ and 0.4 ‰ for Glx and Phe, respectively, Chikaraishi et al., 2009)	Variable depends on AA essentiality (e.g., by average 0.9 ‰ for non‐essential AAs, and 0.1 ‰ for essential AAs, McMahon et al., 2010)
Baseline data applied to TP equation	Species‐specific δ^15^N and its TP (λ), particularly data of primary producer or primary consumer	Fixed (by *β* value 8.4 ‰ and – 3.4 ‰ for terrestrial and aquatic baseline, respectively)	Not applicable
Isotopic baseline	Field sample or literature	Field or lab sample, literature	Field or lab sample, literature
TP estimation	Good	Very good	Limited
Common TP equation	${\text{TP}}_{\text{Bulk}}=\lambda +\left[\frac{\delta^{15}N_{\text{consumer}}‐\delta^{15}N_{\text{baseline}}}{{\text{TDF}}_{\text{Bulk}}}\right]$	${\text{TP}}_{\text{AA}}=1+\left[\frac{\delta^{15}N_{\text{trophic AA}}‐\delta^{15}N_{\text{source AA}}‐\beta }{{\text{TDF}}_{\text{trophic AA}}‐{\text{TDF}}_{\text{source AA}}}\right]$	Not applicable
Dietary breath (herbivory/carnivory)	Yes	Yes	No
Major strength	Quantifies diverse groups niche width, discriminates between trophic positions	Quantifies resources and TP	Good tracers to partition different primary producers
Major limitations	Limited to few numbers of resources, difficulty to find proper basal source	Limits to separate complex resource mixture	Inability to discriminate among trophic levels of prey and its diet
Key references	Layman et al. (2012), Martínez del Rio et al. (2009)	Chikaraishi et al. (2009), Ishikawa (2018), McMahon and McCarthy (2016), Ohkouchi et al. (2017)	Larsen et al. (2009), Larsen et al. (2013), Ohkouchi et al. (2015), Whiteman et al. (2019)

## 2 UNDERSTANDING OF AA ISOTOPE RATIOS VARIANCE FOR ECOLOGICAL STUDIES

### 2.1 N and C isotope variability in consumer AAs

Comparing SIRs in individual AAs is a common step to understand the trophic interactions between consumer and nutritional (or basal) resource. Changes in AA δ^15^N variables during trophic transfer (ΔN) and AA δ^13^C variables (ΔC) are primarily accessed in diverse types of biological (McMahon & McCarthy, 2016; O'Brien, 2015; Pethybridge et al., 2018; Whiteman et al., 2019) and environmental samples (Close, 2019; Ohkouchi et al., 2017). Table 2 presents AA lists grouped by patterns of AA‐SIR variances, as follows:

1. The degree of AA ΔN is generally high or changeless during trophic transfer. For instance, AAs such as glutamic acid/glutamine (Glx) and alanine (Ala), proline (Pro), isoleucine (Ile), leucine (Leu), valine (Val), and aspartic acid/asparagine (Asx) usually become ^15^N enriched during trophic transfer (Table 2). These are commonly called as “trophic” AAs because their δ^15^N values increase during each trophic transfer (Hebert et al., 2016; McCarthy et al., 2007; Nielsen et al., 2015; O’Connell, 2017; Popp et al., 2007). However, several AA δ^15^N changes little with trophic transfer (e.g., ~<1.0‰), commonly observed in phenylalanine (Phe), glycine (Gly), serine (Ser), and lysine (Lys) as listed in McCarthy et al. (2007) and Popp et al. (2007). Since these AA δ^15^N values in consumers resemble those of their diet sources, they are called “source” AAs (McMahon & McCarthy, 2016; O’Connell, 2017; Ohkouchi et al., 2017).
2. The degree of AA ΔC is highly variable or changeless. The variable pattern from (+) to (−) ΔC is commonly detected in Ala, Gly, Ser, and Glx, belonging to non‐essential AAs (NEAAs) that consumers can biosynthesize *de novo* from diverse dietary non‐protein biomolecules (carbohydrates and fats). However, other AA δ^13^C in consumers generally resembles that of their food source with relatively modest alterations during trophic transfer (i.e., ΔC ≈ 0–1‰). The less fractionating patterning is observed mostly in essential AAs (EAAs) encompassing Phe, Lys, methionine (Met), Leu, Ile, threonine (Thr), and Val (Table 2) that consumers cannot biosynthesize *de novo* and should obtain from dietary resources.

**TABLE 2:** Classification of common amino acids (AA) according to their isotope offset patterns between diet and consumers during trophic transfer, which can be triggered by AA nutritional essentiality and metabolism in consumers. Classifications based on data from zooplankton to invertebrates, fish, and mammal

	Carbon		
	Δ^13^C ≈ 0 ‰ (Essential AA)	Δ^13^C ≠ 0 ‰ (Non‐essential AA)	Other AA
Nitrogen			
Δ^15^N ≈ 0 ‰ (Source AA)	Phenylalanine	Glycine^a^	Tyrosine^a^
	Methionine	Serine	
	Lysine		
	Threonine^b^		
Δ^15^N ≠ 0 ‰ (Trophic AA)	Isoleucine	Glutamic acid/Glutamine	
	Leucine	Alanine	
	Valine	Proline	
		Aspartic acid/Asparagine	

^a^ Tyrosine and glycine are conditionally essential AA (Reeds, 2000).

^b^ Threonine offset was not close to 0 (e.g., McMahon et al., 2010).

**FIGURE 2:**
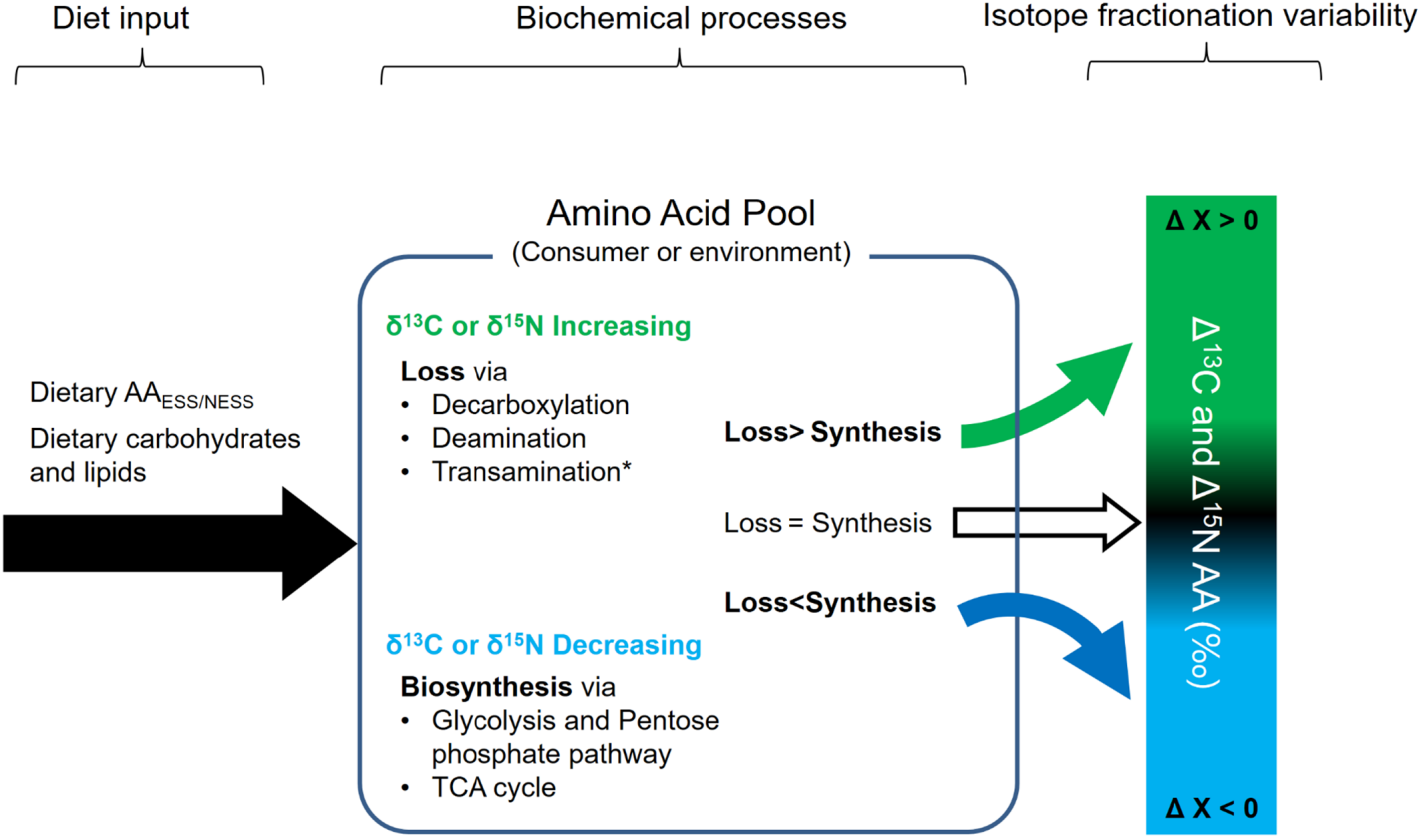
A simplified overview of AA isotope variation in consumer relative to diets. The range of ΔX variations, from (+) to (−), is related to the nutritional essentiality as well as the net balance between losses and biosynthesis via diverse physiochemical processes. (De‐)Transamination and decarboxylation are one of the most common processes related to protein metabolism, which increase δ^15^N and δ^13^C variables of AA, respectively. Biosynthesis process of non‐essential AA originated from non‐protein macronutrients is known to decrease isotope value in a view of substrate–product relation (Fry & Carter, 2019; McMahon & McCarthy, 2016)

**FIGURE 3:**
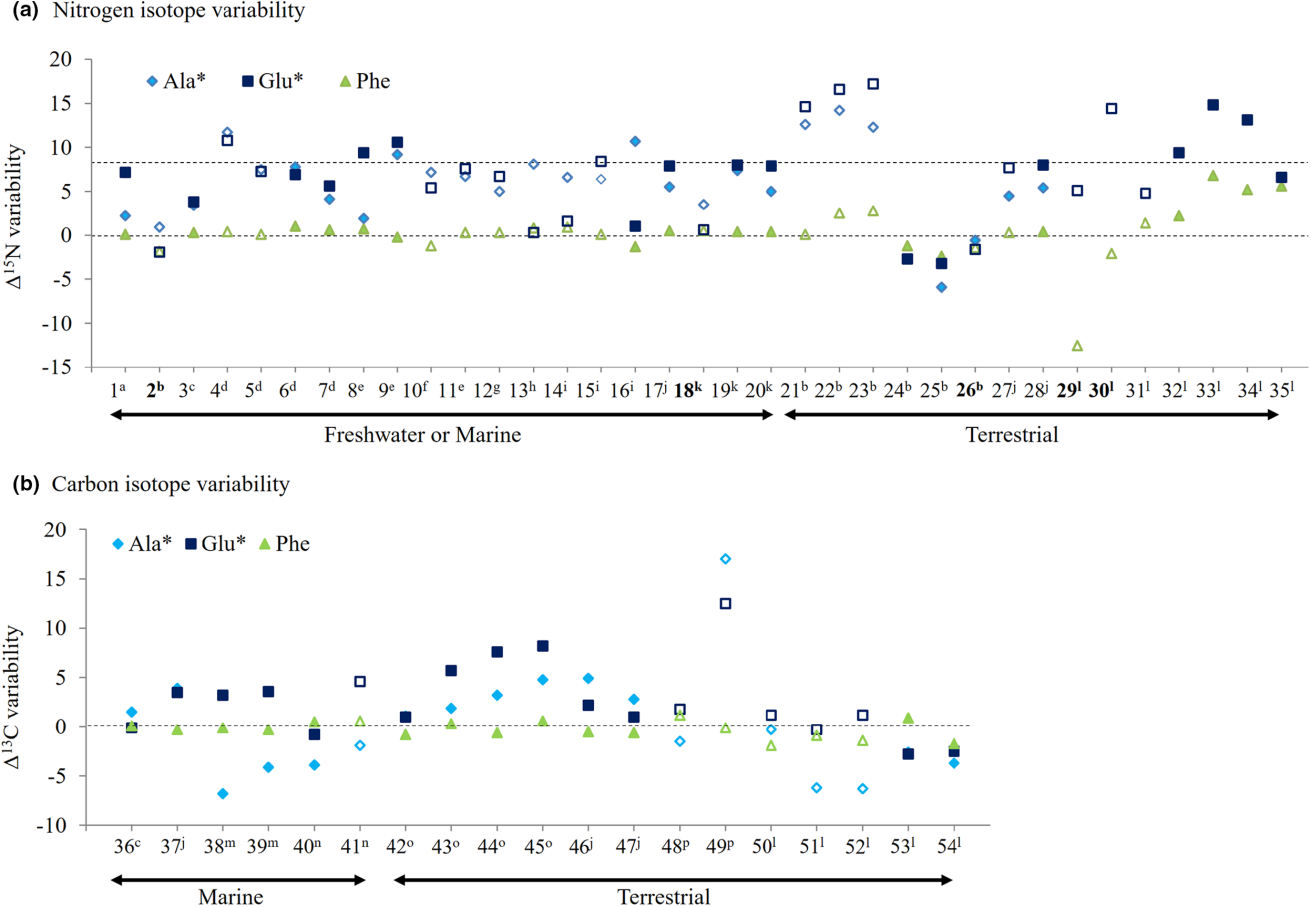
Mean isotope offset variability of AA‐N (A) and AA‐C (B) between diverse consumers and diet obtained from controlled feeding studies. Numbers in X‐axis mean consumer–diet combination pairing (summarized in Table S1). Bolitic font means pairing of consumer and nutritionally poor diet. Diets are grouped to animal‐based protein (marked as closed symbol) vs. producer (including plant, microalgae, fungi, and bacteria)‐based protein (marked as open symbol). Horizontal dashed axis indicates 0 ‰ for source AA and essential AA, and 8 ‰ for trophic AAs (*). Superscripts indicate references ^a^Ishikawa et al. (2014), ^b^Steffan et al. (2015), ^C^McMahon, Polito, et al. (2015), ^d^McMahon, Thorrold, et al. (2015), ^e^Chikaraishi et al. (2009), ^f^Lemons et al. (2020), ^g^McClelland and Montoya (2002), ^h^Gutierrez‐Rodriguez et al. (2014), ^i^Decima et al. (2017), ^j^Takizawa et al. (2020), ^k^Chikaraishi et al. (2015), ^l^Pollierer et al. (2019), ^m^Wang et al. (2019), ^n^Liu et al. (2018), ^o^Newsome et al. (2014), ^p^Jim et al. (2006)

As shown in Figure 2, the overall magnitude of ΔN and ΔC variances in AAs is caused by net results of complex physiological responses. The specified metabolic processes to control N and C variances in AAs are different. For instance, the ^15^N isotope enrichment in consumer AA relative to dietary AA would be connected to active biochemical reactions such as deamination or transamination during N metabolism (Chikaraishi et al., 2007, 2009; McMahon & McCarthy, 2016; O’Connell, 2017; Ohkouchi et al., 2015, 2017), while ^13^C enrichment in consumer could be associated with decarboxylation during C metabolism (Fry & Carter, 2019). Deamination and transamination are N‐involved reactions that convert an amine group (NH_2_) into ammonia (NH_3_^+^) and transfer amine group to keto acids for synthesizing other AAs, respectively. On the other hand, decarboxylation is C‐involved reaction that removes a carboxyl group from a molecule (e.g., AA) and releases carbon dioxide (CO_2_). The N and C involved reactions on AAs, like nearly all physiological reactions, depend selectively on the lighter stable isotope via kinetic fractionation. That is, as the lighter ^14^N or ^12^C‐AAs are preferentially used (or removed) during the metabolic processing, the heavier ^15^N or ^13^C‐AAs would remain more abundantly in consumer body. This is particularly understandable for explaining (highly fractionating) trophic AA δ^15^N patterns listed in Table 2 (McMahon & McCarthy et al., 2016; Ohkouchi et al., 2017). O’Connell (2017) recently proposed that the AA ΔN variability is regulated with the degree of metabolic cycling of an amine group in specific AAs via deamination/transamination processes. As the lighter ^14^N involving NH_2_ group in specific AAs is converted to ammonia and partly removed from metabolic N pool, the heavier ^15^N involving NH_2_ and free ammonia becomes relatively abundant and subsequently incorporated into Glx in the metabolic pool. A greater degree of N exchange between Glx and specific AAs (Glx itself, Ala, Asx, Leu, Ile, Pro, and Val, grouped into trophic AA) is a likely explanation for the ^15^N enrichment patterns in the trophic AA. This is commonly observed in most animal consumers (Figure 3a), from zooplankton (e.g., Choi et al., 2020; Decima et al., 2017; McClelland & Montoya, 2002), amphibians, to birds (e.g., Gomez et al., 2018; McMahon et al., 2015).

However, the heavier isotope enrichment in several AAs (grouped into trophic AAs and NEAAs) is not always detected, and rather the depletion is frequently observed via showing (‐) responses (e.g., 38^m^, 39^n^, 40^n^, 41^n^, 51^l^, and 52^l^ in Figure 3b). Generally, we assume that (‐) Δ responses might relate to biosynthetic process of AAs, as the lighter ^14^N or ^12^C substrates are selectively used to make products (e.g., AAs) than the heavier isotope ^15^N or ^13^C. If AA biosynthesis occurs frequently and is stimulated than anabolic response, AA‐SIR becomes lower than its substrate. In terms of C substrates, intermediates of glycolysis and citric acid cycle, routed from carbohydrates, lipids, and proteins, can be used to synthesize NEAA carbon skeletons. Accordingly, the variability in NEAA ΔC is likely associated with the complex interplay between the relative proportions of dietary macronutrients and the metabolic (catabolic and anabolic) demands within consumer body.

The C isotopic composition of the many AAs in consumers generally resembles that of their food source (Figure 3b, Table S1), with relatively modest alterations during trophic transfer (i.e., ΔC ≈ 0–1‰). The less fractionating patterning is observed mostly in essential AAs (EAAs) (Table 1). This is commonly found in diverse consumers under field sampling and diet‐controlled experiments, such as with sea slugs–sponges (Takizawa et al., 2020), lady bug beetles–aphids (Whiteman et al., 2019), fish–seaweeds (Wang et al., 2019), and penguin–herring (McMahon, Polito, et al., 2015). Since EAA δ^13^C values remain largely invariable during trophic transfer, a food chain encompassing the first consumer (herbivore) to apex predator would have similar EAA δ^13^C values. For instance, in a food chain experiment with the green alga *Chlorella*–copepod *Calanus* (first consumer) and the anchovy *Engraulis* (second consumer), EAA δ^13^C values are unchanged in the herbivorous copepod and alga, the carnivorous anchovy (Liu et al., 2018). Similarly, Phe ΔN of source AAs which belongs to EAA group is changeless (e.g., <1.0‰ in Figure 3) during trophic transfer, although it is still unclear why other source AAs (e.g., Gly and Ser belonging to NEAA). Overall, the fractionation magnitude of the EAAs and the source AAs is not significantly affected by the nutritional content (or nutritional requirement; Jim et al., 2006; McMahon et al., 2010; O'Brien, 2015; Wang et al., 2019) and consumer‐specific N excreting mechanism (McMahon & McCarthy, 2016; Nuche‐Pascual et al., 2021).

### 2.2 Is AA‐SIR variable across spatiotemporal scales?

SIR in compound level is more tightly associated with the exact biomolecules (e.g., AAs), whereas bulk SIR of whole tissue or total protein depends on inter‐molecular isotope differences and the molecular quantities. AA‐SIR values reflect more sensitive physiological responses of consumers to environmental change within local habitats as well as broader geographic scales (Cherel et al., 2019; Choi & Shin, 2021; Laiz‐Carrión et al., 2019; Le‐Alvarado et al., 2021; McMahon et al., 2016; Richards et al., 2020; Smith et al., 2020; Zupcic‐Moore et al., 2017). In fact, AA‐SIR values are controlled by environmental characteristics such as the availability of inorganic substrates, differences in the baseline levels of isotopes, and the physicochemical parameters of local habitats (Decima et al., 2017; Gutierrez‐Rodriguez et al., 2014), or some combination of the three in natural environments (Magozzi et al., 2017, 2021; McMahon et al., 2013). Empirical ecologists expect a considerable troublesome to define the inherent isotope variability of organisms across time, space, and basal sources.

**FIGURE 4:**
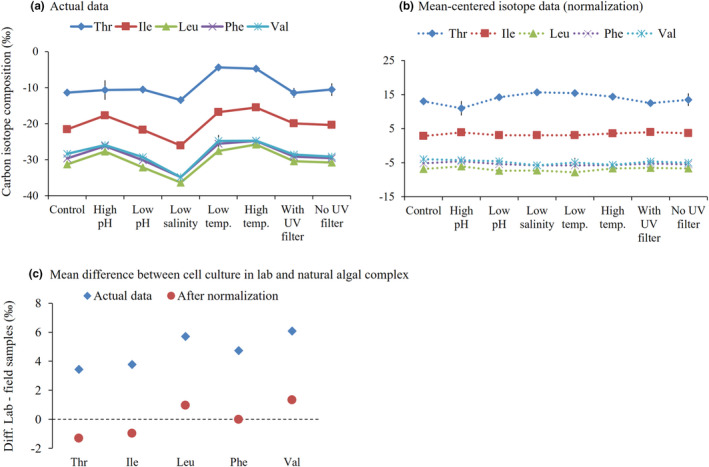
Functioning of normalization to average of multiple essential AAs in autotroph *Thalassiosira weissflogii* under diverse culture treatments from Larsen et al. (2015): (a) Actual data and (b) after isotope value normalization of each AA to the average of five essential AAs, and (c) comparing the variation in essential AA δ^13^C between mean single algal complex and algal complex collected in field (data adopted from Larsen et al., 2013)

Nonetheless, organisms that share similar biosynthetic/metabolic pathways tend to exhibit analogous patterns of isotope fractionation (e.g., Hayes, 2001; Larsen et al., 2009; Scott et al., 2006). It means that overall patterns of AA‐SIR variability are assumed to be highly consistent, although actual AA‐SIR values themselves are significantly different among space and time (Figure 4). The robust AA‐SIR patterns have been presented by placing the isotope variables around the centerline of the average of multiple AAs. The multiple AAs involved are generally the group of essential AAs such as Thr, Ile, Leu, Phe, Val, and Lys. In a case of Larsen et al. (2015) study, the microalga *Thalassiosira* showed a > 6‰ range in overall AA δ^13^C analysis values (Figure 4a), whereas the mean‐centered δ^13^C AA variability reached to within <1‰ (Figure 4b). Moreover, the mean‐centering procedure there showed no clear difference between the cultured microalgal groups (consisting of blue–green algae and diatoms) and the field‐collected microalgal complex (Figure 4c), although their actual AA δ^13^C variables showed dissimilar patterns (> 3‰ difference, on average). In this regard, AA‐SIR patterns detected in the lab‐cultured and green‐house samples can be referred to interpret basal sources in local food webs in nature.

Similar to the AA δ^13^C variability, variances of AA δ^15^N in microalgae are also observed in controlled culture conditions, i.e., growth stage, light regime, herbivory pressure, and N‐ and P‐limited cultured media (Decima et al., 2017; Gutierrez‐Rodriguez et al., 2014). For instance, there is a wide range of AA δ^15^N differences (from <2‰ for Phe to 6‰ for Lys) in both bacteria *Vibrio* and cyanobacteria *Anabaena* cultured with inorganic N sources (N_2_ vs. NO_3_^−^) (Macko et al., 1987). Microalga *Dunaliella* cultured in phosphate (P)‐limited media rather than an N‐limited one showed approximately >3.6‰ higher δ^15^N of Phe, Glx, and Ala (Decima et al., 2017; Gutierrez‐Rodriguez et al., 2014). To factor out the effects of intrinsic spatial heterogeneity, temporal variability, climate change, and nutritional substrates integrated into the C and N isotope baselines in ecosystems, centering the isotope values around the average of the essential AAs (for AA δ^13^C variables) or Phe (for AA δ^15^N variables) has been considered (Hannides et al., 2009; Larsen et al., 2009, 2013; McCarthy et al., 2013). In fact, Phe δ^15^N is particularly well‐known to represent for the N isotope baseline in local environments (Choi & Shin, 2021; Lorrain et al., 2015; Mompean et al., 2016; Sherwood et al., 2011). The offset between Glx δ^15^N and Phe δ^15^N in the microalgae *Thalassiosira*, *Dunaliella*, and *Heterocapsa* became 4.0~5.2‰, regardless of the manipulating culture conditions (Decima et al., 2017; Gutierrez‐Rodriguez et al., 2014). Furthermore, the Glx δ^15^N normalized to Phe δ^15^N in the three microalgae fell within a range similar to that of field‐collected macroalgae as well as cyanobacteria and cultured microalgae, i.e., 3.4 ± 0.9‰ (Chikaraishi et al., 2009, 2015). It implies that AA‐SIR approaches can help us define an isotopic reference of trophic bases and validate basal resources over times and regions in natural environments.

## 3 COMMON USE OF AA δ^15^N AND AA δ^13^C VARIABLES FOR FOOD WEBS STUDIES

### 3.1 Estimating consumer TP

**FIGURE 5:**
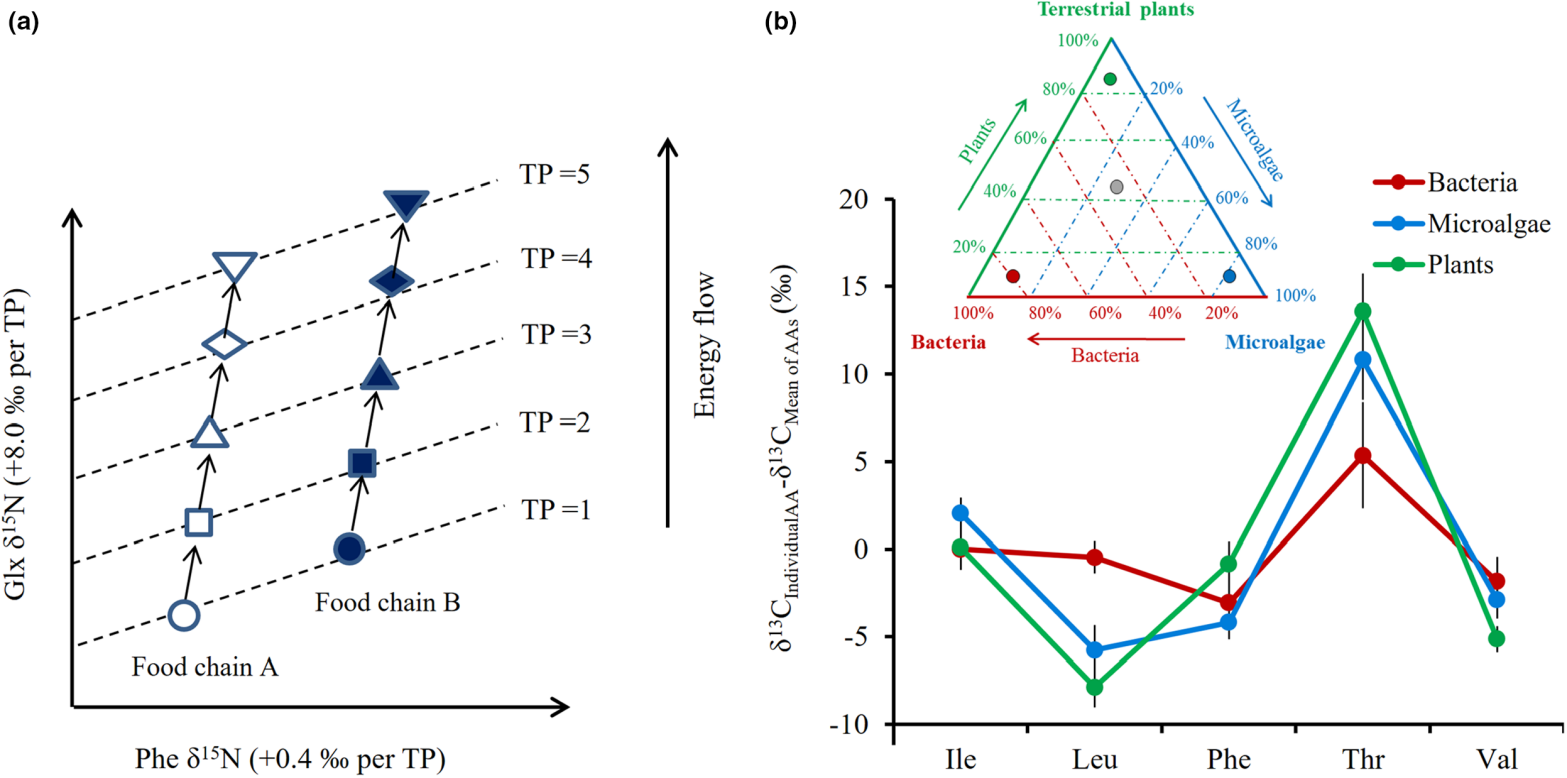
(a) A scheme for describing trophic position using AA‐N variables and (b) example of AA‐C isotope patterns, focused on essential AAs, from cultured species of marine microalgae, terrestrial plants, and heterotrophic bacteria in different nutrient sources, as data were compiled from Larsen et al. (2013). Mean AA isotope values with standard deviation across multiple experimental species are presented. These unique grouping patterns can be applicable as end‐member fingerprints in stable isotope mixing models. Red, blue, and green points within a big triangle indicate consumers which feed mostly on bacteria, microalgae, and terrestrial plants as diets, respectively, while grey point in the middle of triangle is expected to feed mixing of the three sources. The dashed lines help get approximate diet proportion composed of different basal members derived from each organic C resources

Table 1 summarizes the application strategies of AA‐SIR compared with bulk SIR. Based on the consistency in trophic AA and source AA δ^15^N, an AA‐based TP (TP_AA_) equation was developed from a bulk SIR‐based TP equation, as shown in Figure 5a (Chikaraishi et al., 2009). The TP_AA_ is a transformative approach of trophic AA δ^15^N for consumer that is internally indexed to baseline component (or nutritional resource) with source AA δ^15^N variable (Kjeldgaard et al., 2021; McMahon & McCarthy, 2016; Ohkouchi et al., 2017; Ramirez et al., 2021). It is applicable for whole bodies (or any proteinaceous tissue types) from environmental samples to zooplankton and higher trophic‐level fish and apex predators (Cherel et al., 2019; Choi & Shin, 2021; Choi et al., 2020; Germain et al., 2013; Laiz‐Carrión et al., 2019; Mompean et al., 2016; Richards et al., 2020). For obtaining a TP_AA_ estimate, the two reference variables should be involved: the trophic discrimination factors of trophic and source AAs (TDF_AA_), respectively, and the producer‐specific isotope offset of trophic and source AAs from the isotope baseline information as fixed factors, and is followed by:

$$\begin{array}{c} {\text{TP}}_{\text{AA}}=\alpha +\left[\frac{\delta^{15}N_{\text{trophic AA}}‐\delta^{15}N_{\text{source AA}}‐\beta }{{\text{TDF}}_{\text{trophic AA}}‐{\text{TDF}}_{\text{source AA}}}\right]\;(1) \end{array}$$

The δ^15^N_trophic AA_ and δ^15^N_source AA_ represent the nitrogen isotope ratio of trophic AA and source AA analyzed in the consumer; the TDF_trophic AA_ and TDF_source AA_ represent the TDF of each AA; α represents the trophic level of the isotope baseline (if a plant is selected, α = 1; if a primary consumer is involved, α = 2); and the *β* value indicates the δ^15^N difference between the trophic AA and the source AA in the trophic base. Generally, Glx (the canonical trophic AA) and Phe (the canonical source AA) are included in the TP_AA_ estimate. Commonly, a difference between TDF_Glx_ and TDF_Phe_ of 7.6‰ is not variable and generally applied as a constant (Bradley et al., 2015; Chikaraishi et al., 2009; McMahon & McCarthy, 2016; Nielsen et al., 2015). The TDF was found consistently in recent meta‐analyses of wild‐caught marine consumers (Blanke et al., 2017; Nielsen et al., 2015), i.e., 6.6 ± 1.7‰, and consumers under controlled feeding experiments (McMahon & McCarthy, 2016), i.e., 6.2 ± 2.5‰. The *β* value based on Glx‐Phe is also known as fixed factor (Chikaraishi et al., 2009; Ramirez et al., 2021): −8.4 ± 1.6‰ for vascular plants and 3.4 ± 0.9‰ for non‐vascular plants (e.g., aquatic algae). The TP_AA_ estimate achieved 4.8 at maximum, and reached >5.0 for cephalopod *Taningia danae* as well as cetacean *Physeter macrocephalus* in field‐collected samples (Cherel et al., 2019; Troina et al., 2021).

TP_AA_ variance is reliable enough to determine the ecological niche shift of consumers that adapt to local habitats. That is, TP_Glx‐Phe_ variance indicates diet specialization strategies to characterize specialist and generalist feeding habits. In populations collected from 16 ecologically varied habitats, Choi et al. (2020) found that specialist consumers (e.g., pike *Pseudogobio escocinus*) had a narrower range of TP_Glx‐Phe_ magnitude than did generalist fish (e.g., largemouth bass *Micropterus salmoides*). Moreover, obtaining the correct TP_AA_ helps to infer the δ^15^N baseline of local habitats across geographic scales, and ultimately to construct isotope maps to trace migration routes of consumer animals over regions and time periods (Le‐Alvarado et al., 2021; Matsubayashi et al., 2020). Overall, an accurate TP_AA_ estimate enables connecting trophic relations reliably between individual species and, consequently, delineating food web complexity and construction in detail (Bode et al., 2021; Decima & Landry, 2020; Zhang, Tian, et al., 2019).

Determining the TP_AA_ of consumer is frequently generated by Glx and Phe pair, trophic AA and source AA, respectively, and recent studies examined Ala or Pro relative to Phe, and the mean of multiple trophic AA δ^15^N variables relative to the mean of multiple source AA δ^15^N variables. It is very important to obtain proper TDF and *β* parameters, fitted to AA pair, from lab‐/field‐collected or literature data. Such diverse trials produce more realistic TP estimates matched with feeding habits of the consumer widely known (Kjeldgaard et al., 2021; Ledesma et al., 2020; Matthews et al., 2020; Ohkouchi et al., 2017; Ramirez et al., 2021; Troina et al., 2021). For example, in point of δ^15^N_Glx_, protists (heterotrophic microconsumers) show quite similar to phytoplankton (microalga *Dunaliella*) (Decima & Landry, 2020; Decima et al., 2017; Gutierrez‐Rodriguez et al., 2014). This is an unexpected point from their study design that the TP_Glx‐Phe_ of the protists can be like its diet source. In contrast to δ^15^N_Glx_, TP_Ala‐Phe_ in the protists is in the middle between phytoplankton (microalga *Dunaliella*) and metazooplankton (copepods and krill), which is observed consistently in the lab (Decima et al., 2017) and the field (Decima & Landry, 2020), which is close to the realistic trophic relation. It implies that Ala δ^15^N works out effectively in reconstructing the protist involved food webs. Furthermore, Bode et al. (2021) found that the cascading effects from the microplanktonic trophic step enrichment are transferred to a higher trophic level (predator fish), and it makes TP_Ala‐Phe_ 0.5 to 0.6 unit higher than TP_Glx‐Phe_ even for micronecktonic fish. Although the physiological background of the invariable Glx and the variable Ala as trophic AAs in such micro‐sized consumers is still unknown, the comparison of TP_Ala‐Phe_ to the original TP_Glx‐Phe_ estimate could reveal the role of microzooplankton protists in marine food web functioning, which is still poorly understood.

### 3.2 Identifying multiple producers using AA isotope fingerprints

AA‐SIR has been applied for identifying trophic bases (nutritional origins) in local habitats (Table 1). This is due to several empirical evidences that naturally occurring AA δ^13^C variables are unique to distinguish phylogenetically different producers (Larsen et al., 2009, 2013; Scott et al., 2006) (Figure 5a), which is known as “stable isotope fingerprints” (Larsen et al., 2009, 2013). The stable isotope fingerprints based on AAs are related to phylogenetically distinctive characters of producers, such as how to fix inorganic C substrate (e.g., HCO_3_^−^ and CO_2_) and how to synthesize C skeletons of AAs (Brett et al., 2017; Macko et al., 1987; Scott et al., 2006) in diverse routes (e.g., glycolysis, the citric acid cycle, and the pentose phosphate pathway). Currently, ^13^C EAA patterning can separate basal C sources from terrestrial plants, fungi, and heterotrophic bacteria (Larsen et al., 2016; Pollierer et al., 2019; Scott et al., 2006), between seagrass and bacteria (Larsen et al., 2013), and between subtidal kelp (brown macroalga *Laminaria*) and phytoplankton/ephemeral green macroalga (*Ulva*) (Smith et al., 2018). The EAA fingerprint pattern among producer groups can be conceptualized and visualized using dimensionality reduction techniques such as principal component analysis (PCA) and linear discriminant analysis (LDA).

**FIGURE 6:**
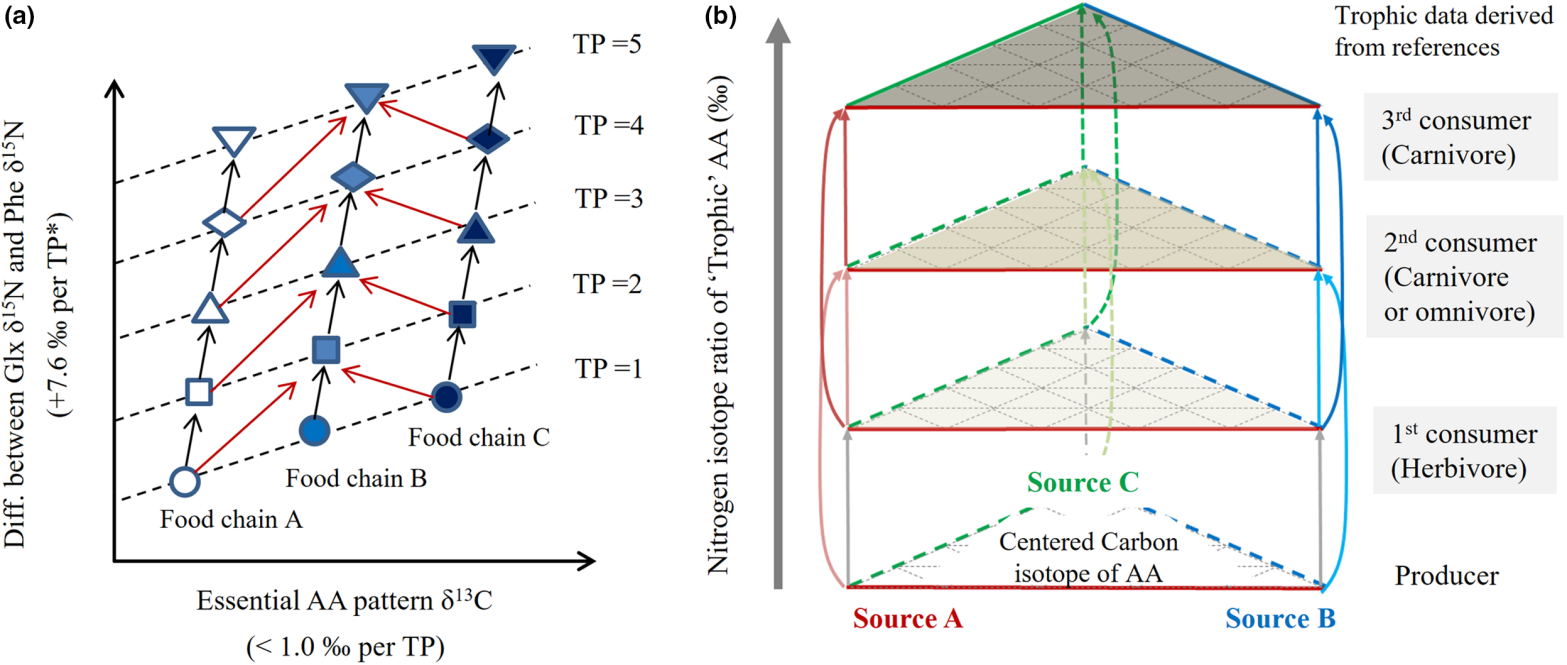
Suggested application of AA‐C and N isotope partitioning method to reconstruct food web structure and to clarify multi‐trophic dietary composition (primary producer or prey). (a) Colored arrows indicate the direction of energy flow from diet to consumer based on AA‐N approaches. * is changeable and affected by specificity of consumer species (McMahon & McCarthy, 2016). Red arrows indicate direction of putative energy flows when collaborating AA‐C and AA‐N approaches. (b) Colored arrows indicate different trophic transfer from sources A, B, and C, respectively, from trophic base members. Straight lines represent trophic relations between consumer (TP belongs to α) and source (TP becomes α‐1), whereas curved lines indicate trophic links with multi‐hierarchy sources (TP could be either α‐1 or even smaller)

Please note that the unique EAA isotope fingerprints can be utilized to estimate their relative contribution in an environment. For instance, if a food wed is composed of three producers (e.g., bacteria, microalgae, and terrestrial plants, which EAA isotope fingerprints are distinct), represented by endpoints of a big triangle, the relative contribution is assessed by stable isotope mixing model. The unique isotope fingerprints in trophic bases are passed to consumers, as actual variables of multiple EAA δ^13^C from nutritional sources are transferred on to consumers. In this regard, the pattern of primary producers in trophic base (TP = 1) is expected to be kept over higher TPs (TP > 1), if there is no extra input of dominant producers. Moreover, the stable isotope fingerprints can also be available with mean‐centered EAA δ^13^C compositions (i.e., difference of AA δ^13^C to average of multiple EAA δ^13^C) to reduce uncertainty related to temporal and regional effect than actual isotope variable (Whiteman et al., 2019). In these regards, the EAA isotope pattern has been a crucial tool to diagnose major basal sources in local food webs (Larsen et al., 2009, 2013, 2016, 2020; Liu et al., 2018).

### 3.3 Identifying nutritional sources from non‐AA components

NEAA δ^13^C variability can reflect physiological responses of consumer to changes in diets or nutrient content in diet source. This is because (1) consumer NEAA δ^13^C is tightly linked to the isotopic values of diverse C source pools derived from dietary carbohydrates, fats, and proteins (Jim et al., 2006; McMahon et al., 2010; O'Brien, 2015; Wang et al., 2019). It supports the physiological response that NEAAs are mainly synthesized from sugars, monoglycerides, free fatty acids, and AAs through the digestion of the three macronutrients. Several controlled feeding experiments on various consumers (rat, pig, moth, and fish) showed that the Ala δ^13^C value in animals (pig and butterfly moth) depends on the δ^13^C value of dietary non‐protein component (carbohydrate) derived from C_3_ (−28‰) or C_4_ plants (−12‰), which is not directly associated with Ala δ^13^C in diets (Jim et al., 2006; McMahon et al., 2010; Newsome et al., 2011, 2014; O'Brien et al., 2002). Even at high protein levels, controlled feeding experiments with varying diet proportions of isotopically distinct protein/carbohydrates (Jim et al., 2006; Newsome et al., 2011) and protein/lipids (Newsome et al., 2014) showed that the NEAA δ^13^C variability is closely associated with non‐protein dietary sources. Similarly, feeding experiments with rats fed diets with varying proportions of protein and lipids with distinct isotope values (Newsome et al., 2014) supported that C in lipid is used to biosynthesize C skeletons in NEAA.

Moreover, the NEAAs Δ^13^C variances from (+) to (−) responses (see the Section 2.1) help reveal about how consumers adapt to the shift in diet quality and nutritional needs with increasing size or age (O'Brien, 2015; Whiteman et al., 2019). For instance, O'Brien et al. (2002) found that NEAA δ^13^C compositions in eggs of a hawkmoth depend on C_3_‐ and C_4_‐based sugars offered to their adult moth in feeding experiments, which are isotopically different as −25‰ vs. −11 ‰, and proving that allocates nectar C to produce NEAAs for egg production. NEAA δ^13^C compositions in fish are also significantly affected by digestibility of carbohydrate (Wang et al., 2019) as well as the macronutrient composition in meals, i.e., plant type (carbohydrate‐based diets) vs. normal fishmeal (fat‐/protein‐based diets) (McMahon et al., 2010). Thus, variations in NEAA δ^13^C values appears to have an important ecological implication, particularly true for animals that often undergo seasonal or spatial changes in macronutrient availability (Magozzi et al., 2021), and also for animals that consume lipid‐rich prey, which contributes ~25–50% of the C source of marine animals (Newsome et al., 2014), or carbohydrate‐rich fruit, which contributes ~50–90% of the C source for migratory birds (Gomez et al., 2018).

## 4 INTERPRETATION OF AA‐SIR FOR DEFINING TROPHIC TRANSFER BETWEEN GREEN AND BROWN FOOD WEBS

There is considerable interest to reveal ecological functions of green‐based source (mainly composed of non‐detrital OM by photoautotrophs) and brown‐based source (mostly detrital OM) for consumer community. However, current reviews on AA‐SIR applicability scope focus more to describe the trophic ecology for small and large metazoan consumers (McMahon & McCarthy, 2016; Whiteman et al., 2019). This review focuses on the potential of AA‐SIR to reveal how detrital OM is involved in food web functioning as a consumer and producer in diverse ecosystems using AA‐SIR.

### 4.1 Defining trophic interactions of consumers with green‐ as well as brown‐based resource

Since consumer AA δ^15^N is tightly associated with diet (or basal sources) AA δ^15^N (Topic 1 in Table 3), the consumer SIR can reveal the change in diet (or resource) shifts (Figure 5a). This application is particularly useful when the isotope variables between the basal sources are significantly distinguishable (Phillips et al., 2014). Several studies tested whether AA δ^15^N variables themselves (Ishikawa, 2018; Naito et al., 2016) as well as whether generated TP_AA_ values (Bode et al., 2021; Decima & Landry, 2020; Kubiak et al., 2021) could evaluate the relative importance of dual dietary sources for consumers in the stable isotope mixing model. For instance, Ishikawa et al. (2014) used two isotope variables (Glx and Phe δ^15^N) to build two source mixing models and to assess the relative importance of periphytonic algae (>60%) over leaf litter (a detrital component) from herbivorous flies and carnivorous fish in a stream ecosystem. Owing to the effect of vascularization on *β* values (e.g., −8.4 ± 1.6‰ for vascular plants and 3.4 ± 0.9‰ for non‐vascular plants) in TP_Glx‐Phe_, seasonal variances in TP_AA_ estimate proposed that Pleistocene caribou foraged on marine algae relative to terrestrial plants during periods of snow cover (Kubiak et al., 2021).

**TABLE 3:** Use of AA‐C and N isotope application and its relevant research topics at individual, species, community, and ecosystem level

Topic	Species and samples examined	Habitat type	Key literatures	AA isotopes
Topic 1: Identifying trophic relations in simple or multi‐trophic food chains				
In direct consumer–prey relation, confirming AA‐based TP index of consumer relative to dietary items	Diverse organisms from micro‐ to bigger sized consumers (e.g., zooplankton, earthworm to fish)	Aquatic/Terrestrial	McClelland and Montoya (2002), Chikaraishi et al. (2009), Chikaraishi et al. (2014), Tsuchiya et al. (2018), Liew et al. (2019), Pollierer et al. (2019)	N
Checking TP and food chain length of consumer relative to diet	Simulating food chain with four trophic levels: algae, water fleas, guppies, and bluegill sunfishes	Aquatic	Bowes and Thorp (2015)	N
Suggesting Ala as canonical trophic AA, rather than Glx	Simulating food chain with three trophic levels: algae, protist (microzooplankton Ciliate and dinoflagellate), and copepod (mesozooplankton *Calanus*)	Aquatic	Decima et al. (2017)	N
Calculating TP and food chain length can be assessed to explain ecological structure of shallow water hydrothermal vent ecosystem	Vent‐associated POM, chemoautotrophic population, zooplankton, epibenthic crustaceans, and vent‐obligate crab *Xenograpsus*	Aquatic	Chang et al. (2018)	N
Detecting cyanobacteria blooms and heterotrophic microbes in local trophic base, supporting a feeding mode for mesozooplankton.	Zooplankton (100–300 μm and >300 μm size groups) by vertical tows	Aquatic	Loick‐Wilde et al. (2019)	N & C
Expecting phytoplankton effects as other diet source for zooplankton, not seston, without direct sampling	Zooplankton *Acartia* and diet source seston	Aquatic	Nielsen and Winder (2015)	N
Tracking AA source for structuring mesozooplankton community: N_2_‐fixing autotroph vs. chemoautrotrophic bacteria (or archaea) sources	POM and mesozooplankton (Cladocerans, copepod *Pseudo*‐ and *Paracalanus*) in surface, suboxic, and bottom water	Aquatic	Eglite et al. (2018)	N
Uncovering trophic chains in soil food webs	Primary consumers (spring tails, oribatid mites), predator (spiders and gamasid mites), and basal resources (living plants, fungi, and bacteria)	Terrestrial	Pollierer et al. (2019)	N & C
Defining dietary origins (e.g., aquatic vs. terrestrial C source) based on essential AAs for large animals	Animals (e.g., Green turtles, deep Sea coral, cave fish, and pig)–Basal C resources of local habitats	Aquatic/Terrestrial	Arthur et al. (2014), Schiff et al. (2014), Liew et al. (2019), Ferrier‐Pagès et al. (2021)	C
Topic 2: Distinguishing potential resources and Characterizing the source contribution in community levels				
Proving unique EAA patterns among primary producers	Microalgae, seaweed, seagrass, bacteria, and cyanobacteria	aquatic/terrestrial	Scott et al. (2006), Larsen et al. (2009), Larsen et al. (2013)	C
Proving any differences in N sources in autotrophic producers	Cyanobacteria vs. eukaryotic algae	Aquatic	McCarthy et al. (2013)	N
Defining food webs structure based on metabolic pathways of amino groups and useful to complex food webs structure, where heterogeneous resources (aquatic vs. terrestrial) are mixed.	Macroinvertebrates, fishes, and their potential food sources (periphyton and leaf litter of terrestrial C3 plants)	Aquatic	Ishikawa et al. (2014)	N
Elaborating TP estimate via considering source contribution (by distinguishing seagrass and particulate organic matter) and adopting proper beta variables	Community members from producers to consumer, such as seagrass to fish community in riverine community and aquatic/terrestrial sources to cave fish in cave ecosystems	Aquatic	Choi et al. (2017), Liew et al. (2019)	N
Explaining transport and deposition of sediment grains from river to coastal zone due to distinguishing marine and terrigenous material	Marine sediment, terrestrial debris, river SPM, estuarine diatoms, phytoplankton, and zooplankton	aquatic	Keil and Fogel (2001)	C
Describing N source, transformation, and food web structure	Plankton tow, sinking POM, and DOM	Aquatic	McCarthy et al. (2007), Mompean et al. (2016), Yamaguchi and McCarthy (2018), Romero‐Romero et al. (2020)	N
	Deep sea zooplankton, sinking POM in small vs. large particles	Aquatic	Demopoulos et al. (2019), Romero‐Romero et al. (2020)	N
Tracing carbon sources as trophic base	Potential food sources and their consumers	Aquatic/Terrestrial	Ziegler and Fogel (2003), Scott et al. (2006), Larsen et al. (2009), Larsen et al. (2013), Vokhshoori et al. (2014), Thorp and Bowes (2017)	C
Topic 3: Exploring nutrient cycle in geographic scales				
Describing N source, transformation, and food web structure	Plankton, sinking POM, and DOM	Aquatic	McCarthy et al. (2007), Mompean et al. (2016), Yamaguchi and McCarthy (2018), Romero‐Romero et al. (2020)	N
Tracing C relationship between POM and DOM	POM (0.7–10um), bacterial fraction (0.2–0.7um), and DOM	Aquatic	Ziegler and Fogel (2003)	C
Topic 4: Diagnosing changes in ecological niche in consumer				
Comparing diverse C sources of resource utilization for fish community in local sites	Consumer fish and its local prey items (e.g., zooplankton, seaweeds, coral reef tissues)	Aquatic	McMahon et al. (2016), Larsen et al. (2020)	C
Indicating animal community changes in response to anthropogenic activity	Consumer community changes	Aquatic/Terrestrial	Ruiz‐Cooley et al. (2017), Chua et al. (2021), Wilkinson et al. (2021)	N
Determination of diet source, foraging area, and trophic position for mobile top predators	Four penguin groups (three species), tuna, bowhead whale, sperm whale, sunfish, squids, Weddell seal	Aquatic	Lorrain et al. (2009), McMahon, Polito, et al. (2015), Madigan et al. (2016), Huckstadt et al. (2017), Zupcic‐Moore et al. (2017), Cherel et al. (2019), Laiz‐Carrión et al. (2019), Phillips et al. (2020), Le‐Alvarado et al. (2021)	N & C
Topic 5: Physiological changes in consumer to variable diet quality				
Refining trophic discrimination factor (TDF) throughout multiple AAs in terms of C	Omnivorous fish *Fundulus* and *mice Mus* and herbivorous *Butterfly* grown in controlled nutritional qualities	Aquatic/Terrestrial	O'Brien et al. (2002), McMahon et al. (2010), Newsome et al. (2014)	C
Quantifying carbohydrate carbon used by the gut microbiome to synthesize essential AAs and assimilation in host consumer	Host animal (Mice *Mus*, Fish *Nile*, and Earthworm *Enchytraeus)* and gut microbiome	Aquatic/Terrestrial	Newsome et al. (2011), Larsen et al. (2016), Newsome et al. (2020)	C
Checking AA synthesis routed from dietary carbohydrates and lipids	Fish with animal‐ and vegi‐based meal	Aquatic	Bloomfield et al. (2011), McMahon et al. (2015b), Nuche‐Pascual et al. (2018)	N
Confirming changeless TDF, not affected by satiation/starvation	Dobsonfly *Protohermes* under nutritional status (satiation vs. starvation)	Aquatic	Ishikawa et al. (2014)	N
Dietary routing/biosynthesis of non‐essential AA by juvenile crab growth period, Specifying isotopic fractionation in non‐essential AA and essential AA due to major diet source	Crab *Callinectes*, snail, meiofauna, zooplankton, detritus, *Spartina*	Aquatic	Fantle et al. (1999)	C
Specifying AA isotopic fractionation in terms of δ^15^N and δ^13^C among tissue types	Migratory songbird and insects and fruits as diets	Terrestrial	Gomez et al. (2018)	N & C
Detecting AA carbon and nitrogen isotope fractionation relevant to (in)organic substrates	Bacteria and microalga cultured in organic and inorganic substrates	Aquatic	Macko et al. (1987)	N & C
Specifying AA isotopic fractionation in terms of δ^15^N and δ^13^C	Polyp and skeleton in deep sea coral, TP calculation (consumer)	Aquatic	McMahon et al. (2018)	N & C
	Detecting AA‐SIR of flower and leaf parts from tree plants	Terrestrial	Takizawa et al. (2017)	N

Although dead OM (i.e., detritus combined with living bacteria and/or fungi) is more heterogeneous and dynamic than living OM, AA^15^N analysis is useful to determine the trophic links of “brown” detritus‐based food webs (Topic 2 in Table 3). From a certain inter‐trophic enrichment between consumer detritivores and dead OM, AA‐SIR can assess and quantitatively estimate the role of detritus complex as food web component. For instance, abyssal detritivores (Riekenberg et al., 2021; Romero‐Romero et al., 2021) and micro‐/mesozooplanktons (Doherty et al., 2021) showed higher AA δ^15^N composition and higher TP relative to nutritional resources as derived from detrital OM (i.e., zooplankton fecal pellet). Furthermore, it is available to examine the trophic connections between the brown food chains and the green food chains. For instance, Steffan et al. (2017) conducted controlled feeding trial experiment that the decomposed plants and prey animals (surrounded by bacteria or fungi) regarded as the brown diets, respectively, were offered to herbivores and carnivores for 1 month. In perspective of TP_Glx‐Phe_ estimate, the brown (decomposed) foods were about half a TP up compared to fresh diets, which is observed consistently for both herbivores and carnivores; this suggests that the characteristics of detritus are subsequently transferred to increase the TP of detritus‐feeding consumers (i.e., nutritional absorption from the detritus into consumer). Thus, TP_AA_ can provide a perspective for reconstructing the complex food web structure derived from the mixture of brown and green sources for macrosized consumers in natural environments.

AA δ^15^N approach is powerful to detect the transformation of detrital OMs dominated by microbes (bacteria) of marine environments in context of biogeochemical processing (Topic 3 in Table 3). For instance, in a middle‐depth water column where photosynthetic autotrophs rarely exist (water depth >250 m and deeper), source AA ^15^N in fractions of large particles (> 53 µm, assuming a short microbial alternation) are depleted than the one in smaller particles (0.7–53 µm, assuming a long microbial alternation) (Romero‐Romero et al., 2020). Such source AA δ^15^N results in middle‐depth waters, which suggest that smaller‐sized fractions contain more decomposed organic compounds as a result of active bacterial degradation compared to larger‐sized fractions that are relatively fresh (Yamaguchi & McCarthy, 2018). Similarly, Hannides et al. (2020) found a ~4‰ difference between the two OM fractions, especially in summer. In a microscale, ultra‐filtered particulate OM (a large particle fraction) in ocean water column contains slightly low AA δ^15^N relative to dissolved OM fractions (Yamaguchi & McCarthy, 2018).

Not only AA‐SIR value itself but also a proxy (the ∑V index, called total “heterotrophic AA resynthesis”) can express the magnitude of microbial (bacterial) activity, which describes the δ^15^N variability of multiple trophic AAs from the average of δ^15^N values of multiple trophic AAs (e.g., Leu, Val, Ile, Ala, Pro, and Glx), as originally proposed by McCarthy et al. (2007). The defined ranges of ∑V values in phytoplankton and metazoans are 0–1 and 1–2, respectively, whereas ∑V values >2 indicate detrital OM (brown resource) with an increasing proportion of degrading algae and zooplankton (Arthur et al., 2014; Hannides et al., 2020; McCarthy et al., 2007). Notably, ∑V values are greatly increased in smaller dissolved OM compared to particulate OM (Yamaguchi & McCarthy, 2018). As food web base components, detrital (non‐living) OM and its small molecular size can be linked to its microbial reactivity and degradation rate in middle‐depth water; these might be more active and fresher near the ocean surface (Doherty et al., 2021; Stücheli et al., 2021).

With developing analytical method (i.e., coupling of polar and non‐polar column in gas chromatography system), inclusion of the δ^15^N of multi‐source AAs, Phe, and Met could be an option in the isotope mixing model (Ishikawa et al., 2018). This approach can be effective because (1) inclusion of multiple isotope variables can handle with distinguishing more diverse sources (e.g., Choi et al., 2017; Ishikawa et al., 2014) and (2) the source AAs provide a more direct proxy indicating the δ^15^N base relative to the local habitats (e.g., offshore vs. nearshore and/or benthic vs. pelagic) rather than the consumer‐specific metabolic effect on isotope variability (Quillfeldt & Masello, 2020).

### 4.2 Quantifying the relative importance of green vs. brown sources in consumer diets

As actual variables of multiple EAA δ^13^C from nutritional sources are transferred on to consumers (Figure 6b), EAA isotope fingerprints are also passed on to consumers. In this aspect, EAA isotope pattern in consumer can determine major basal sources via conducting stable isotope mixing models (Larsen et al., 2009, 2013, 2016, 2020; Liu et al., 2018). Thus, the EAA fingerprint approach is regarded as a reliable tool to diagnose the basal EAA sources and its relative proportion in food webs, particularly even when direct sampling of trophic base components is difficult or unknown.

EAA isotope fingerprints of higher TP consumers can elucidate the basal C origins in green sources (autotroph such as microalgae, cyanobacteria, and plants‐relevant OM) vs. brown sources (non‐autotroph such as bacteria‐ and fungi‐originated OM) for sessile (Ferrier‐Pagès et al., 2021; Wall et al., 2021) and mobile macrobenthos (Smith et al., 2018, 2020), and fish as tertiary (or upper) TP consumers in aquatic ecosystems (Larsen et al., 2020; Thorp & Bowes, 2017). Recently, the applicability of EAA δ^13^C fingerprint has been extended to distinguish two brown groups, heterotrophic bacteria and fungi, which both are well‐known potential decomposers in underground soil and sediment. Since EAA δ^13^C fingerprints in heterotrophic bacteria are notably different from those in fungi (Larsen et al., 2013; Scott et al., 2006), they can determine major C sources for detritivores such as earthworms and microarthropods (Larsen et al., 2016; Pollierer & Scheu, 2021). Furthermore, EAA isotope fingerprints have been applied to environmental samples, such as sediment, water column, and soil samples. For instance, Brett et al. (2017) evaluated the primary C sources on algal food resources relative to terrestrial plant‐derived OM and heterotrophic bacteria in riverine ecosystems. Thus, EAA fingerprints approach has been broadly applied from consumer bodies to water columns and sediments to characterize actual diets and/or basal C resources in local food webs.

## 5 LIMITATIONS OF AA‐SIR IN IDENTIFYING FOOD WEB STRUCTURES AND ECOSYSTEM FUNCTIONING

### 5.1 AA δ^15^N approach: Beyond the robust ΔN patterns in consumers

The certain magnitude of the isotope variability in AAs during trophic transfer should be crucial to describe the true ecological niche of consumers and their resource reliance (McMahon & McCarthy, 2016; Nuche‐Pascual et al., 2021). This variability in the canonical trophic AA Δ^15^N is generally recognized as, on average, >7.8‰ in Glx for diverse trophic levels of consumers (Bradley et al., 2015; Chikaraishi et al., 2009, 2014; Steffan et al., 2017). It can further vary in the specific consumers in aquatic ecosystems (Figure 3a).

The trophic AAs Δ^15^N values can be affected by diet quality (e.g., protein insufficient foods, marked as 2^b^, 4^d^, and 18^k^ in Figure 3a). The possible physiological reaction of consumers is that intake of protein insufficient foods may increase the AA recycling and, thus, result in ^15^N enrichment in consumer AAs (Hoen et al., 2014; McMahon & McCarthy, 2016; Nuche‐Pascual et al., 2021). For instance, fish *Fundulus* fed a plant‐based diet showed high Glx Δ^15^N and Ala Δ^15^N than with an animal‐based diet of pelletized commercial fish meal (a clam‐ and squid‐based diet) (average Glx: 10.8‰ vs. 5.6‰; Ala: 11.7‰ vs. 4.1‰, marked as 4^d^, 6^d^, and 7^d^ Figure 3a). However, Chikaraishi et al. (2015) reported that amphibian tadpoles of common toads (*Bufo bufo*) fed with boiled rice (a plant diet) showed significantly low trophic AAs Δ^15^N than when fed commercial fish pellets and bloodworms (an animal‐based diet). Of smaller consumer organisms, copepod *Calanus* fed with dinoflagellate *Oxyrrhis*, the dinoflagellate *Oxyrrhis* fed with alga *Dunaliella* (Gutierrez‐Rodriguez et al., 2014), and ciliate *Favella* fed with alga *Heterocapsa* (Decima et al., 2017) showed small variances in Glx (<1‰) but large variability in Ala of trophic AAs (> 8‰). In extreme cases of prolonged lack of food (for months), free‐ranging mammals (elephant seals) showed higher AA δ^15^N values compared to successful hunting periods, when their metabolic response was to transform AAs into energy sources during the fasting period (Lübcker et al., 2020).

Although relatively high variance of Glx and/or Ala Δ^15^N is commonly reported in aquatic consumers, this is not usually the case in terrestrial ecosystems (Figure 3a). For instance, Steffan et al. (2015) reported negative Δ^15^N in Glx and Ala when insects and fungi on a plant‐based diet showed somewhat lower isotope values than their dietary AA (i.e., soy–wheat); however, similar consumer groups have shown enriched ^15^N AA isotope values relative to animal‐based diets (i.e., army worms). Similarly, detritivores and decomposers (e.g., *Heteromurus*) showed variable Phe Δ^15^N in aquatic consumers, from −12.5‰ (feeding on plants) to −2.1‰ (feeding on bacteria) (Pollierer et al., 2019) (Figure 3a). The depletion of AA ^15^N (particularly, Thr as one of the source AAs in Table 2) is also reported in omnivore rat which feeds on high protein diets compared to one with low protein diets (Fuller & Petzke, 2017), but more studies are needed to understand how Thr δ^15^N variations are affected by diet quality (Whiteman et al., 2021). Such unexpected fractionation patterning in terrestrial consumers (detritivores and omnivores) is not fully understood, and can suggest a mismatch of nutrient content to nutritional demand from a consumer (e.g., Pollierer et al., 2019).

### 5.2 AA δ^15^N approach: Beyond the robust ΔN patterns in primary producers

For vascular plants, the δ^15^N offsets between Glx and Phe values are observed as −8.4 ± 1.6‰ (Chikaraishi et al., 2011), which was close to the −9.3 ± 1.6‰ for woody plants (Kendall et al., 2019). However, the range of the δ^15^N offset in plants that have a true vascular system, root, and leaf is highly dependent on their morphology (reviewed in Ramirez et al., 2021). For instance, several herbaceous plants (grasses) show the Glx–Phe difference as −5.8 ± 2.1‰ contrast to tree plants (Kendall et al., 2019). Moreover, δ^15^N offsets between Glx and Phe showed photosynthesis organelles (leaves) with approximately −8.4‰ vs. non‐photosynthesis organelles (flowers) with approximately <3.5‰ (Takizawa et al., 2017). Potentially, these different patterns between plant types and/or plant tissues are due to higher lignin content, especially in the woody plants which involve Phe in their phenylpropanoid pathways to produce lignin and other phenolic secondary metabolites, leading to isotopic fractionation and enrichment of the remaining Phe pool (Kendall et al., 2019). Possibly, the dynamic δ^15^N offsets between Glx and Phe values from terrestrial plants might not be observed in non‐vascular producers (e.g., seaweeds) that are composed of namely stipe, holdfast, and blade and lack of flowering part. This can lead to erroneous consumer TP values in certain ecosystems (soil, stream, and riverine food webs) involving terrestrial plants (Ramirez et al., 2021).

### 5.3 Beyond dietary EAA δ^13^C estimates: Effects of the microbiome

There are exceptional cases wherein the EAAs Δ^13^C between consumers and their diets are substantial (Figure 3b), ranging from 2.5‰ to >7.5‰ (Table S1). Such phenomena are reported even in designed feeding studies with diverse consumers such as rats (Newsome et al., 2020), enchytraeid worm (Larsen et al., 2016), and coral (Ferrier‐Pagès et al., 2021). The mismatch of δ^15^C between consumer EAAs and dietary EAAs is usually interpreted as the nutritional contribution of de novo synthesized microbes (i.e., by the gut microbes) (Arthur et al., 2014; Larsen et al., 2016; Newsome et al., 2011, 2014, 2020). This is usually a feature in consumers adapted to live off nutritionally imbalanced diets, such as detritus, wood, and other low protein diets with low digestibility (Arthur et al., 2014; Ayayee et al., 2016; Larsen et al., 2016; Newsome et al., 2011; del Rio et al., 2009). Larsen et al. (2016) also reported by using EAA δ^13^C fingerprints that enchytraeids (oligochaetes) in Arctic peatlands derived 80% of their EAAs gut symbiotic bacteria EAAs. 16S rRNA sequencing of enchytraeids from a subsequent feeding trial found that EAA microbiome contribution to host and the taxonomic composition of the gut microbiome were significantly associated with the diets’ nutritional contents, such as the proportions of starches relative to fibers. A similar finding was found for mice living off protein‐deficient diets with low digestibility (Newsome et al., 2020). By comparing Δ^13^C values and the relative abundance of specific gut microbial taxa, Newsome et al. (2020) concluded that gut microbiome (e.g., *Firmicutes*) made a greater contribution of Val (~60%) to host than Phe (<6%) when the mice were fed protein‐deficient and hardly digestible diets. These findings emphasize that for some animals, the microbiome can play an important role in supplementing macronutrients that are otherwise insufficient in the diet.

## 6 COLLABORATION OF N AND C‐SIR IN AAs: FUTURE STUDY

There are increasing trends of dual analysis of N and C isotope variables in AAs, but generally their main roles in ecological contexts are highly differentiated. For instance, AA‐N assessment as a reliable tool has commonly elucidated trophic pathways in detail, while AA‐C assessment improves our understanding about multiple basal C resources in trophic energy flow as well as tracking biosynthetic C transfer (Table 1). We assume that change in the AA‐C isotope composition is inherently different from that of the AA‐N variable. However, the potential of collaborating AA‐N and C‐SIR applicability to characterize population niche and community structure is still less concerned, and thus we suggest some possible applications in the following section.

### 6.1 Suggestion 1. Proxies for assessing the ecological niche of a focal species

A δ^13^C‐δ^15^N bi‐plot, relying on bulk analysis, is powerful to describe food web structure and population niche in a local habitat, but there are difficulties in comparing different populations (or communities) spatially and temporally. This is because bulk isotope ratios in the basal resources often show spatial and temporal variations, directly reflected to those of consumer (Shipley & Matich, 2020). Furthermore, information of basal resources (primary producers) and their sampling in local food webs are not always reliable. As explained in Section 2.2, AA‐SIR variables are relatively more robust to temporal and spatial variations in local habitats than bulk δ^13^C‐δ^15^N variable, informing basal N or C sources (belonging to trophic bases) by source AA and essential AA, respectively. Accordingly, we believe that AA δ^15^N and AA δ^13^C compositions in the bi‐plot shown in Figure 6a might help comparing temporally and spatially different populations (or communities) as well as informing trophic links more effectively. For instance, basal sources (multi‐producers and OM) can be well separated in the lowest part and consumers are in relatively higher positions within the AA‐SIR plot. As the direction changes, the size of the organism is larger and AA isotope composition is generally higher, so this plot helps visualize trophic structure and trophic dependence in complex environments in high resolution. In fact, Cherel et al. (2019) firstly approached collaborative AA‐SIR bi‐plot to explore foraging ecology of several cephalopod species, from long‐term collection over 20 years and different sites, and wherein the X‐axis was δ^13^C of Gly and the Y‐axis was the relative trophic position (RTP = δ^15^N_Glx_ – δ^15^N_Phe_ of consumers). The two isotopic indices help characterize the trophic distances among the consumer species, and ultimately disentangle species‐specific isotopic niches. In this way, community‐wide isotope parameters such as food (trophic) chain length, trophic base diversity, and ecological niche space can also be replaced by AA‐SIR, which has been originally approached by bulk isotopic values (e.g., Layman et al., 2007). A large isotopic niche length or width (area) of a consumer might imply a wider range of diet choice. When the isotope niches between consumers are not distinct in local habitats, we might interpret it as overlap of consumer niches.

### 6.2 Suggestion 2. Estimating complex diet composition of omnivores

To uncover the real diet compositions of consumers in local habitats, it is first necessary to disentangle heterotrophic diets and multiple basal sources. The best known δ^13^C fingerprinting approach, discussed here, involves characterizing the source mixtures composed of primary producers as well as bacteria to infer the trophic pathways of the basal C resources for general consumers (Figure 6b). However, δ^13^C fingerprinting works poorly to distinguish diet contributions in mixtures of multi‐trophic diet sources, since the C isotope values of EAAs in a plant, a plant‐eating prey animal, and a consumer (e.g., an omnivore and its diet items) cannot be separated. Instead, AA δ^15^N measurement is a possible solution that accounts for multi‐trophic sources and consumers. Combining AA δ^15^N and δ^13^C compositions in AAs can be an option in a stable isotope mixing model (Figure 6b). The traditional approach of mixing bulk C and N isotopes allows the ecologist to calculate the proportional contributions of two or four food resources in a consumer diet (Phillips & Koch, 2002). Instead, the new model can be fitted with AA δ^13^C and δ^15^N variables, accommodating more diverse food resources by including the numbers of more useful isotope variables. Consequently, estimating the proportional contributions in multiple diets of heterotrophic‐level prey animals has important implications for interpreting the ecological functions of consumers that are surrounded by complex diet sources in nature.

## 7 CONCLUSION

In conclusion, the traditional SIR approach can be a valuable tool for source determination, but still struggles with environmental variation, limited numbers of sources determination, and ambiguous trophic transfers. Consequently, bulk SIR is still difficult to address trophic interactions, food web dynamics, and ecological processes objectively and logically in local habitats. Future work should utilize compound‐specific SIR approach to describe ecological and biogeochemical processes, particularly if sampling of trophic base is too difficult or unknown to get enough sample size. To extend AA‐SIR applicability in the future, analytic technique should be improved to produce more reliable data of certain essential AAs (histidine, tryptophan, and methionine), which are substantially underestimated in ecological/geochemical context. Interpretation of the AA‐SIR pattern with underlying knowledge of AA metabolism allows us to properly place consumers among the diet candidates, and accordingly bring to bear ecologically meaningful ecological proxies such as TP estimates, trophic chain lengths, and dietary source portions, and ultimately viewing food web structures and ecosystem functions more clearly.

## AUTHOR CONTRIBUTIONS

**Hee Young Yun:** Conceptualization (lead); Writing – original draft (lead); Writing – review & editing (lead). **Thomas Larsen:** Methodology (supporting); Writing – original draft (supporting); Writing – review & editing (supporting). **Bohyung Choi:** Conceptualization (supporting); Writing – original draft (supporting). **Eun‐Ji Won:** Investigation (supporting); Writing – original draft (supporting); Writing – review & editing (supporting). **Kyung‐Hoon Shin:** Project administration (lead); Writing – original draft (supporting).

## CONFLICT OF INTEREST

The authors declare that they have no conflict of interest.

## Supporting information

Table S1

## Data Availability

The data in this article were obtained from publically available publications through Web of Science. Variables for drawing Figure 3 are listed in Supporting Information.
