# Supplementary material for: Amino acid nitrogen and carbon isotope data: Potential and implications for ecological studies
Source: Ecol Evol. 2022 Jun 2;12(6):e8929. doi: 10.1002/ece3.8929 (PMC9163675; doi:10.1002/ece3.8929)
Supplement: Supplementary file 1 — Table S1 [file ECE3-12-e8929-s001.docx]

**Supporting information**

**Supporting Table.** Trophic fractionation factors (mean Δ^15^N_C-D_ and Δ^13^C_C-D_ in ‰) in several AAs for terrestrial, aquatic and marine consumers in response to dietary resources via controlled feeding experiments. Resource type marked with ‘§’ indicates plant-based diets, nutritionally poor, for carnivores and omnivores.

| Pair | Species | Resource type | Habitat | Ala* | Glu* | Pro* | Phe# |
| --- | --- | --- | --- | --- | --- | --- | --- |
| **AA-N base** | | |  |  |  |  |  |
| 1a | Dobsonfly *Protohemrmes*  larva | Chironomid larvae | Aquatic | 2.2 | 7.2 | ud | 0.1 |
| 2b | Shrimp *Branchinecta* | Soy-wheat diet^§^ | Aquatic | 0.9 | -1.9 |  | -1.8 |
| 3c | Penguin *Pygoscelis* | Herring | Aquatic | 3.4 | 3.8 | 5.2 | 0.3 |
| 4d | Fish *Fundulus* | Plant-based diet | Aquatic | 11.7 | 10.8 | 7.3 | 0.4 |
| 5d | Fish *Fundulus* | Bio-Vita | Aquatic | 7.5 | 7.3 | 7 | 0.1 |
| 6d | Fish *Fundulus* | Clam | Aquatic | 7.8 | 6.9 | 6.7 | 1 |
| 7d | Fish *Fundulus* | Squid | Aquatic | 4.1 | 5.6 | 6.6 | 0.6 |
| 8e | Fish *Paralichthys* | Rotifer *Brachionus* | Aquatic | 1.9 | 9.4 | 5.9 | 0.7 |
| 9e | Fish *Sebastes* | Rotifer *Brachionus* | Aquatic | 9.2 | 10.6 | 5.9 | -0.2 |
| 10f | Turtle |  | Aquatic | 7.2 | 5.4 | 5.5 | -1.2 |
| 11e | Rotifer *Brachionus* | Alga *Chlorealla* | Aquatic | 6.7 | 7.6 | 7.9 | 0.3 |
| 12g | Rotifer *Brachionus* | Alga *Tetracelmis* | Aquatic | 5 | 6.7 | 4 | 0.3 |
| 13h | Dinoflagellate *Oxyrrhis* | Alga *Dunaliella* | Aquatic | 8.1 | 0.3 | 3.5 | 0.8 |
| 14i | Ciliate *Favella* | Alga *Heterocapsa* | Aquatic | 6.6 | 1.6 |  | 0.9 |
| 15i | Copepod *Calanus* | Alga *Thalassiosira* | Aquatic | 6.4 | 8.4 |  | 0.1 |
| 16i | Copepod *Calanus* | Dinoflagellate *Oxyrrhis* | Aquatic | 10.7 | 1 |  | -1.3 |
| 17j | Sea slug *Hypselodoris* | Sponge *Halichondria* | Aquatic | 5.5 | 7.9 |  | 0.5 |
| 18k | Bufo | Boiled rice^§^ | Aquatic | 3.5 | 0.6 |  | 0.5 |
| 19k | Bufo | Fish　meal (Plant+Animal) | Aquatic | 7.4 | 8 |  | 0.4 |
| 20k | Bufo | Worm (Animal rich type) | Aquatic | 5 | 7.9 |  | 0.4 |
| 21b | Insect *Dermestes* | Armyworm | Terrestrial | 12.6 | 14.6 |  | 0.1 |
| 22b | Fungi *Beauveria* | Armyworm | Terrestrial | 14.2 | 16.6 |  | 2.5 |
| 23b | Fish *Poecilia* | Armyworm | Terrestrial | 12.3 | 17.2 |  | 2.8 |
| 24b | Insect *Tribolium* | Soy-wheat diet | Terrestrial | -2.7 | -2.7 |  | -1.2 |
| 25b | Fungi *Flammulina* | Soy-wheat diet | Terrestrial | -5.9 | -3.2 |  | -2.4 |
| 26b | Mice *Mus* | Soy-wheat diet^§^ | Terrestrial | -0.6 | -1.6 |  | -1.4 |
| 27j | Ladybug beetle | Aphid | Terrestrial | 4.5 | 7.7 |  | 0.3 |
| 28j | Green lacewing | Armyworm | Terrestrial | 5.4 | 8 |  | 0.4 |
| 29l | Insect *Heteromurus* | Lime leaves^§^ | Terrestrial |  | 5.1 |  | -12.5 |
| 30l | Insect *Archegozetes* | Lime leaves^§^ | Terrestrial |  | 14.4 |  | -2.1 |
| 31l | Insect *Parasteatoda* | *Heteromurus* fed on lime leaves | Terrestrial |  | 4.8 |  | 1.4 |
| 32l | Insect *Heteromurus* | Bacteria^§^ | Terrestrial |  | 9.4 |  | 2.2 |
| 33l | Insect *Sinella* | Bacteria | Terrestrial |  | 14.8 |  | 6.8 |
| 34l | Insect *Archegozetes* | Bacteria | Terrestrial |  | 13.1 |  | 5.2 |
| 35l | Insect *Parasteatoda* | *Heteromurus* fed on bacteria | Terrestrial |  | 6.6 |  | 5.6 |
| ***AA-C base*** | | |  | Ala* | Glu* | Pro* | Phe# |
| 36c | Penguin *Pygoscelis* | Herring | Aquatic | 1.5 | -0.1 | 0.1 | 0.1 |
| 37j | Sea slug | Sponge | Aquatic | 3.9 | 3.5 |  | -0.3 |
| 38m | Fish *Salmo* | Fishmeal+corn starch | Aquatic | -6.8 | 3.2 | 2.8 | -0.1 |
| 39m | Fish *Salmo* | Fishmeal+*Palmaria* | Aquatic | -4.1 | 3.6 | 3.1 | -0.3 |
| 40n | Fish *Engraulis* | Copepod *Calanus* | Aquatic | -3.9 | -0.8 | 1.4 | 0.5 |
| 41n | Copepod *Calanus* | Alga *Chlorella* | Aquatic | -1.9 | 4.6 | 4.1 | 0.6 |
| 42o | Mice *Mus* | Casein+Lipid5% | Terrestrial | 1.1 | 1 | 2.8 | -0.8 |
| 43o | Mice *Mus* | Casein+Lipid15% | Terrestrial | 1.9 | 5.7 | 3.2 | 0.3 |
| 44o | Mice *Mus* | Casein+Lipid25% | Terrestrial | 3.2 | 7.6 | 5.1 | -0.6 |
| 45o | Mice *Mus* | Casein+Lipid40% | Terrestrial | 4.8 | 8.2 | 7.4 | 0.6 |
| 46j | Ladybug beetle | Aphid | Terrestrial | 4.9 | 2.2 |  | -0.5 |
| 47j | Green lacewing | Armyworm | Terrestrial | 2.8 | 1 |  | -0.6 |
| 48p | Rat | Formulated diet (C_3_ Protein+C_4_Carbohydrate | Terrestrial | -1.5 | 1.8 | 2.9 | 1.2 |
| 49p | Rat |  | Terrestrial | 17 | 12.5 | 4.8 | -0.1 |
| 50l | Insect *Heteromurus* | Variable diet resource prey from lime leaves, yeast, lupine and fungi to bacteria | Terrestrial | -0.3 | 1.2 | 1.8 | -1.9 |
| 51l | Insect *Sinella* |  | Terrestrial | -6.2 | -0.3 | 2.3 | -0.9 |
| 52l | Insect *Archegozetes* |  | Terrestrial | -6.3 | 1.2 | -0.4 | -1.4 |
| 53l | Insect *Stratiolaelaps* |  | Terrestrial | -2.6 | -2.8 | -2.2 | 0.9 |
| 54l | Insect *Parasteatoda* |  | Terrestrial | -3.7 | -2.5 | -1.6 | -1.7 |

Superscripts indicate references ^a^Ishikawa et al. (2014), ^b^[Steffan et al. (2015)](#_ENREF_13), ^C^[McMahon, Polito, Abel, McCarthy, and Thorrold (2015)](#_ENREF_9), ^d^[McMahon, Thorrold, Elsdon, and McCarthy (2015)](#_ENREF_10), ^e^[Chikaraishi et al. (2009)](#_ENREF_1), ^f^[Lemons, Lewison, Seminoff, Coppenrath, and Popp (2020)](#_ENREF_6), ^g^[McClelland and Montoya (2002)](#_ENREF_8), ^h^[Gutierrez-Rodriguez, Decima, Popp, and Landry (2014)](#_ENREF_4), ^i^[Decima, Landry, Bradley, and Fogel (2017)](#_ENREF_3), ^j^[Takizawa et al. (2020)](#_ENREF_14), ^k^[Chikaraishi, Steffan, Takano, and Ohkouchi (2015)](#_ENREF_2), ^l^[Pollierer et al. (2019)](#_ENREF_12), ^m^[Wang, Wan, Krogdahl, Johnson, and Larsen (2019)](#_ENREF_15), ^n^[Liu, Luo, and Cai (2018)](#_ENREF_7), ^o^[Newsome, Wolf, Peters, and Fogel (2014)](#_ENREF_11), ^p^[Jim, Jones, Ambrose, and Evershed (2006)](#_ENREF_5).

**References**

Chikaraishi, Y., Ogawa, N. O., Kashiyama, Y., Takano, Y., Suga, H., Tomitani, A., . . . Ohkouchi, N. (2009). Determination of aquatic food-web structure based on compound-specific nitrogen isotopic composition of amino acids. *Limnology and Oceanography-Methods, 7*, 740-750. doi:10.4319/lom.2009.7.740

Chikaraishi, Y., Steffan, S. A., Takano, Y., & Ohkouchi, N. (2015). Diet quality influences isotopic discrimination among amino acids in an aquatic vertebrate. *Ecology and Evolution, 5*(10), 2048-2059. doi:10.1002/ece3.1491

Decima, M., Landry, M. R., Bradley, C. J., & Fogel, M. L. (2017). Alanine delta(15) N trophic fractionation in heterotrophic protists. *Limnology and Oceanography, 62*(5), 2308-2322. doi:10.1002/lno.10567

Gutierrez-Rodriguez, A., Decima, M., Popp, B. N., & Landry, M. R. (2014). Isotopic invisibility of protozoan trophic steps in marine food webs. *Limnology and Oceanography, 59*(5), 1590-1598. doi:10.4319/lo.2014.59.5.1590

Jim, S., Jones, V., Ambrose, S. H., & Evershed, R. P. (2006). Quantifying dietary macronutrient sources of carbon for bone collagen biosynthesis using natural abundance stable carbon isotope analysis. *British Journal of Nutrition, 95*(6), 1055-1062. doi:10.1079/bjn20051685

Lemons, G. E., Lewison, R. L., Seminoff, J. A., Coppenrath, C. M., & Popp, B. N. (2020). Nitrogen isotope fractionation of amino acids from a controlled study on the green turtle (Chelonia mydas): expanding beyond Glx/Phe for trophic position. *Marine Biology, 167*(10), 149. doi:10.1007/s00227-020-03745-3

Liu, H. Z., Luo, L., & Cai, D. L. (2018). Stable carbon isotopic analysis of amino acids in a simplified food chain consisting of the green alga Chlorella spp., the calanoid copepod Calanus sinicus, and the Japanese anchovy (Engraulis japonicus). *Canadian Journal of Zoology, 96*(1), 23-30. doi:10.1139/cjz-2016-0170

McClelland, J. W., & Montoya, J. P. (2002). Trophic relationships and the nitrogen isotopic composition of amino acids in plankton. *Ecology, 83*(8), 2173-2180. doi:10.1890/0012-9658(2002)083[2173:tratni]2.0.co;2

McMahon, K. W., Polito, M. J., Abel, S., McCarthy, M. D., & Thorrold, S. R. (2015). Carbon and nitrogen isotope fractionation of amino acids in an avian marine predator, the gentoo penguin (Pygoscelis papua). *Ecology and Evolution, 5*(6), 1278-1290. doi:10.1002/ece3.1437

McMahon, K. W., Thorrold, S. R., Elsdon, T. S., & McCarthy, M. D. (2015). Trophic discrimination of nitrogen stable isotopes in amino acids varies with diet quality in a marine fish. *Limnology and Oceanography, 60*(3), 1076-1087. doi:10.1002/lno.10081

Newsome, S. D., Wolf, N., Peters, J., & Fogel, M. L. (2014). Amino Acid delta C-13 Analysis Shows Flexibility in the Routing of Dietary Protein and Lipids to the Tissue of an Omnivore. *Integrative and Comparative Biology, 54*(5), 890-902. doi:10.1093/icb/icu106

Pollierer, M. M., Larsen, T., Potapov, A., Bruckner, A., Heethoff, M., Dyckmans, J., & Scheu, S. (2019). Compound-specific isotope analysis of amino acids as a new tool to uncover trophic chains in soil food webs. *Ecological Monographs, 89*(4), 24. doi:10.1002/ecm.1384

Steffan, S. A., Chikaraishi, Y., Currie, C. R., Horn, H., Gaines-Day, H. R., Pauli, J. N., . . . Ohkouchi, N. (2015). Microbes are trophic analogs of animals. *Proceedings of the National Academy of Sciences of the United States of America, 112*(49), 15119-15124. doi:10.1073/pnas.1508782112

Takizawa, Y., Takano, Y., Choi, B., Dharampal, P. S., Steffan, S. A., Ogawa, N. O., . . . Chikaraishi, Y. (2020). A new insight into isotopic fractionation associated with decarboxylation in organisms: implications for amino acid isotope approaches in biogeoscience. *Progress in Earth and Planetary Science, 7*(1). doi:10.1186/s40645-020-00364-w

Wang, Y. M. V., Wan, A. H. L., Krogdahl, A., Johnson, M., & Larsen, T. (2019). C-13 values of glycolytic amino acids as indicators of carbohydrate utilization in carnivorous fish. *Peerj, 7*. doi:10.7717/peerj.7701
